# Preliminary Validation of the Malayalam‐version of the Cognitive Change Index

**DOI:** 10.1002/alz.088442

**Published:** 2025-01-09

**Authors:** Marnina B Stimmel, Ayers Emmeline, Joe Verghese, Erica Weiss, Laura Rabin

**Affiliations:** ^1^ Montefiore Medical Center, Bronx, NY USA; ^2^ Albert Einstein College of Medicine, Bronx, NY USA; ^3^ Department of neurology, Division of Cognitive and Motor Aging, Albert Einstein College of Medicine, New York, NY USA; ^4^ Department of Neurology, Division of Cognitive and Motor Aging, Albert Einstein College of Medicine, New York, NY USA; ^5^ Brooklyn College of the City University of New York, Brooklyn, NY USA

## Abstract

**Background:**

With the worldwide geriatric population steadily increasing, identifying older adults in need of treatment for cognitive difficulties is imperative. Limited specialty resources makes it particularly important to identify which individuals would most benefit from further evaluation and treatment, including in countries where dementia care is limited, such as India. Self‐report measures are timely and cost effective tools that can assist providers in identifying individuals at risk of cognitive decline. The Cognitive Change Index (CCI), a 20‐item measure of self‐perceived cognitive functioning, has been validated in English and Spanish. In the present study, we present preliminary findings on the translated, Malayalam‐version of the CCI.

**Method:**

500 Malayalam‐speaking older adults (mean age = 68.8[SD = 5.4]; 62% male; mean education = 9.6 years[SD = 3.5]) completed the CCI, Addenbrooke’s Cognitive Examination‐III (ACE‐III) and Geriatric Depression Scale‐30 item (GDS‐30) as part of the Kerala Einstein Study, an ongoing longitudinal community‐based study of pre‐dementia syndromes in Kerala India. The CCI was translated and back‐translated for accuracy. Means and SDs were calculated for various baseline demographics. Pearson correlation and t‐tests (and cohen’s d for effect size) were used to compare the CCI to demographic variable and to GDS‐30 and ACE‐III performances.

**Result:**

Higher endorsement on the CCI was strongly correlated with lower ACE‐III performances (r(498) = ‐.66, p <.001), and weakly correlated with higher GDS‐30 scores (r(498) = .33, p <.001). The CCI was not correlated with age or education. While men endorsed significantly higher CCI scores (mean = 37.7; SD = 16.9) compared to women (mean = 34.8; SD = 15.3), t(498) = ‐1.925, p = .027, the effect size was small (Cohen’s d = ‐.178) and the difference not clinically meaningful. When gender and GDS‐30 were controlled, the correlation between CCI and ACE‐III remained strong (r(498) = ‐.66, p <.001).

**Conclusion:**

Preliminary data showed confirmatory performance of the Malayalam‐version of the CCI in identifying individuals at risk for cognitive impairment (i.e., lower performances on a cognitive screener), and was not meaningfully influenced by age, gender, or education. Although additional validation is needed, this preliminary analysis suggests that the CCI could be a useful self‐report measure for providers to use with Malayalam speaking older adults to ascertain possible cognitive impairment.

